# An empirical study on purchase intention for home-based care services and its influencing factors in Liaoning Province, China

**DOI:** 10.3389/fpubh.2026.1821105

**Published:** 2026-06-30

**Authors:** Qin Sen, Liu Duoduo

**Affiliations:** 1 School of Humanities and Management, Ningxia Medical University, Yinchuan, China; 2 School of Economics and Management, Ningxia University, Yinchuan, China

**Keywords:** aged, home care services, intention, logistic models, Shapley value

## Abstract

**Background:**

Home-based care services are becoming the predominant model of older adult care in China, with a vast market potential. However, challenges such as insufficient service demand and hindered industrial development persist, requiring urgent attention to the transformation of older adults’ service needs into consumption behaviors.

**Methods:**

Using survey data collected from 14 cities in Liaoning Province, China, by a research team from Ningxia University, binary logistic regression and Shapley value decomposition were employed to investigate older adults’ purchase intention (i.e., willingness to pay for services, not actual utilization behavior) for home-based care services and its influencing factors.

**Results:**

The findings reveal that older adults with younger age, higher education level, strong individual needs, sufficient resource endowments, or diversified perceptions of caregiving responsibilities had a higher likelihood of purchasing services. Community resources emerged as the most critical factor influencing purchase intention. Of the service categories, the intention to purchase life care services ranked highest, followed by healthcare services, while information advisory services had the lowest intention.

**Discussion:**

To enhance the quality of life for older adults and optimize service provision, the study recommends: (1) expanding the supply scope of home-based care services and promoting the development of community-embedded care service facilities; (2) improving the income levels of older adults to strengthen their purchasing capacity; and (3) optimizing the supply structure of service programs based on differentiated needs.

## 1 Introduction

Since China officially became an aging society in 2000, its trend of population aging has only intensified (1). Active steps to address population aging have thus been incorporated into the national strategic framework. The “14th Five-Year Plan for the Development of National Aging Affairs and Care Service System” explicitly demands the continuous expansion of “care service for older people that covers both urban and rural areas, benefits all citizens, is balanced and reasonable, and of high quality and efficiency.” Home-based care services for older people, which rely on communities to provide various care resources to households, primarily provide medical care, daily living assistance, and spiritual comfort, among other types of support. This model is influenced by China’s traditional concepts of care for older people and the realities of China’s modern social development, making it a key strategy for actively responding to the issue of population aging. Survey results from the China Consumer Association in 2022 show that 88.93% of older people choose the home-based care service model (2). Despite this vast customer demand, the development of such services remains relatively weak, mainly due to a mismatch between service supply and demand. This has led to a lack of recognition and willingness to purchase home-based care services for older people, as well as the hampered development of related industries. Studying older people’s real needs and purchase intentions for home-based care services and exploring the underlying influencing factors is thus of significant research value.

This study focuses on older adults’ purchase intention (i.e., willingness to pay for services) rather than actual purchasing behavior, as intention is a key predictor of demand and real-world behavior data are unavailable in our survey. Although previous studies have identified various factors influencing older adults’ purchase intention for home-based care services (3), most have focused on isolated individual characteristics without a systematic, multi-dimensional framework. Furthermore, few have used large-scale, multi-city data to compare the relative contribution of community resources versus personal resources, and even fewer have examined purchase intentions separately for different service types. To fill these gaps, the present study uses survey data from 3,562 older adults across 14 cities in Liaoning Province, applies an extended Andersen behavioral model that incorporates older adult care concepts, and employs a combination of regression analysis and decomposition techniques to quantify the importance of four factor groups. The findings can inform targeted policy interventions to align service supply with heterogeneous demand.

Against this background, this study aims to answer the following questions. First, what is the current purchase intention of older Chinese people toward home-based care services? Which service items do older people most need? What factors influence older people’s purchase intention toward care services? What factors may influence the types of service items older people purchase? In the context of promoting active aging in China, answering these questions can deepen our understanding of the care service needs of older people, thereby enabling more effective allocation and utilization of care resources. Ensuring that resources meet the most urgent needs and providing a basis for the government to formulate relevant policies can contribute to the construction of a more comprehensive social support system.

## 2 Literature review and research hypotheses

Research on home-based care services for older people began in the 1970s, with early studies primarily focusing on the service needs of older people (4). These studies explored the reasons why existing service frameworks failed to meet service demands and proposed corresponding strategies for improvement (5). Subsequent scholars gradually realized that there is a significant issue of transforming service demand into actual consumption behavior (6), and began to focus on older people’s purchasing propensity for services, attempting to explore the underlying influencing factors involved. Su et al. found that older people’s economic status is the most important factor affecting their purchase of services, including their income situation, support from children, local government subsidies, etc. (7) Van argued that individual characteristics of older people, such as age, education level, marital status, mobility, etc., significantly affect their service needs and purchase of services (8). Luppa argued that regional factors influence older people’s willingness to purchase services, manifested in noticeable regional differences and urban–rural disparities (9). Overall, studies to date have primarily focused on specific individual factors as core independent variables in isolation, and a systematic model of influencing factors has not yet been developed. This may have resulted in the omission of variables or the introduction of bias, resulting in larger discrepancies in the research results and weakening the rigor and scientific nature of the research conclusions.

In 1968, American scholar Ronald Andersen constructed the “Health Services Utilization Behavior Model”, known as the traditional Andersen model, to analyse the influencing factors of health services utilization. This model has significant application value in the field of health care, especially in health policy formulation, medical service planning, and health management (10). It posits that an individual’s health services utilization behavior is influenced by three key factors: predisposition characteristics, service demand, and enabling resources. Predisposition characteristics refer to the traits of individuals in the population using the services; service demand denotes an individual’s perceived demand for service items, while enabling resources involves the individual’s ability to access services and the availability of related resources (11). The traditional Andersen behavior model is a powerful tool for analysing and predicting health services utilization behavior. It has been continuously revised and expanded over the past 50 years, forming a more robust theoretical framework that adapts to new research and practical needs.

This study expands the scope of the traditional Andersen model to the study of older people’s willingness to purchase home-based care services. There are three significant reasons for doing so. First, health care services and home-based care services for older people are, by nature, quasi-public goods, with service recipients making purchase decisions based on their own needs and consumption capacity. Second, under the policy background of promoting the integration of medical care and care, health care services constitute home-based care services for older people and thus occupy an important position. Third, the long-term development and revision of the Andersen model has led to it being widely applied to research on demand for, and utilization of, care services, and it has been found to have a high degree of theoretical model fit (12). Based on research results to date, the theoretical framework of the traditional Andersen model suggests that the influencing factors of older people’s willingness to purchase care services can be summarized into three categories: basic characteristics, individual needs, and resource conditions.

The first of these, “basic characteristics,” are the socio-demographic characteristics that can influence older people’s willingness to purchase care services. These are primarily their gender, education level, marital status, and living arrangements. In terms of gender, some studies have found that older males are more willing to purchase care services than females (13). In terms of age, some studies have found that younger people are more willing to purchase care services than older people (14). In terms of education level, some scholars have found that older people with higher education generally have a higher willingness to purchase care services than those with middle and low education levels (15). In terms of marital status, some studies have shown that the spouses of older people play a specific role as a caregiver, which may lead to a lower willingness of married older people to purchase care services than that of unmarried older people (16). In terms of living arrangements, the willingness to purchase care services is stronger for older people living alone (17).

The second, “individual needs,” are the physiological and psychological needs that can stimulate older people’s willingness to purchase care services. Specifically, in terms of physiological needs, studies have shown that older people’s health status directly and significantly affects their willingness to purchase services. Older people with disabilities, semi-disabilities, or chronic diseases generally have a stronger willingness to purchase care services. In terms of psychological needs, older people with a strong sense of loneliness and anxiety are more willing to purchase care services than those without (18). In summary, based on the relationship between the individual needs of older people and their willingness to purchase home-based care services, this paper proposes the first set of research hypotheses:

*Individual Need (Physiological) Hypothesis H1a*: The physiological needs of older people affect their willingness to purchase home-based care services: the stronger their physiological needs, the stronger their willingness to purchase services.

*Individual Need (Psychological) Hypothesis H1b*: The psychological needs of older people affect their willingness to purchase home-based care services: the stronger their psychological needs, the stronger their willingness to purchase services.

Finally, “resource conditions” are the social, family, and individual resources that can affect the willingness of older people to purchase care services. Specifically, social resources are manifested in the accessibility of home-based care services for older people. Hu et al. found that when social service resources are abundant and easily accessible, enabling older people to conveniently obtain care services, their willingness to purchase such services is stronger (19). Family resources comprise the material or daily care support provided by offspring. Kenneth et al. found that the stronger the support from adult children, the stronger the willingness of older people to purchase services (20). Individual resources are primarily represented in older people’s income level. A large number of studies have shown that older people’s income level has a significant positive impact on their willingness to purchase care services (21). In summary, based on the relationships linking the resource conditions of older people to their willingness to purchase home-based care services, this paper proposes the second set of research hypotheses:

*Resource Condition (Social) Hypothesis H2a*: Older people’s social resources affect their willingness to purchase home-based care services: the richer their social resources, the stronger their willingness to purchase services.

*Resource Condition (Family) Hypothesis H2b*: Older people’s family resources affect their willingness to purchase home-based care services: the richer their family resources, the stronger their willingness to purchase services.

*Resource Condition (Individual) Hypothesis H2c*: Older people’s individual resources affect their willingness to purchase home-based care services: the richer their individual resources, the stronger their willingness to purchase services.

Moreover, in the social and cultural context of Chinese society, the concept of care may also influence older people’s willingness to purchase services. The traditional Andersen model neglects this element, thereby weakening its adaptability for research in China. According to Ajzen’s Theory of Planned Behavior, attitudes and subjective norms are key factors that influence individuals’ behavioral intentions. Su et al. found that the concept of family older adult care is deep-rooted in Chinese culture, and many older people are unwilling to pay for care services outside the family. In contrast, older people with more diverse concepts of older adult care have a stronger willingness to purchase care services. In summary, based on the relationship between care concepts of older people and their willingness to purchase home-based care services, this paper proposes the third set of research hypotheses:

*Care Concept Hypothesis H3*: Older people’s care concepts affect their willingness to purchase home-based care services, in that older people who rely more on family and children for care have a weaker willingness to purchase than those who rely more on older adult self-care.

In summary, this study extends the traditional Andersen model in three specific ways. First, while most prior applications have focused on service utilization, we adapt the model to examine purchase intention, a conceptually distinct but policy-relevant outcome. Second, we incorporate older adult care responsibility perception as an additional dimension, acknowledging the cultural context of filial piety in China. Third, we introduce Shapley value decomposition to quantitatively compare the relative explanatory power of four factor groups (basic characteristics, individual needs, resource conditions, and care concepts). These extensions do not constitute a new theoretical construct but rather provide a more nuanced empirical framework for understanding the drivers of purchase intention in the Chinese home-based care setting. Based on this extended framework, we propose three sets of hypotheses concerning individual needs (H1a, H1b), resource conditions (H2a, H2b, H2c), and older adult care concepts (H3).

## 3 Methods

### 3.1 Data source

The data for this study were obtained from a large-scale survey conducted by the research team in 14 cities of Liaoning Province from 2021 to 2024. The survey aimed to explore the implementation of home-based care services for older people in Liaoning Province; the current study is a sub-project of that larger survey. Liaoning Province was chosen for several key reasons. First, it has a high degree of population aging, with statistics showing that more than 25.7% of the population is aged 60 years or above, indicating a significant demand for home-based care services. Second, the province has a relatively high level of economic development, and thus has the financial capacity to support the construction and improvement of its home-based care service system. Third, the provincial government of Liaoning has shown a strong willingness to support care services, enacting multiple policies to foster the development of the home-based care service system there and achieving several practical results. Fourth, the moderate number of cities in Liaoning permits a survey that covers a range of sizes and types of urban areas, enhancing the applicability and representativeness of the research findings.

This study used a multi-stage stratified cluster sampling method, targeting community-dwelling individuals aged 60 and above. The survey process comprised the following steps. First, the sample size for each city was allocated based on population size (see Table 1). Second, sampling communities were identified using systematic random methods. Third, cluster sampling was used to select 10 older adult individuals from each community. The survey was conducted via a questionnaire, with surveyors reading questions aloud when necessary, ensuring the completeness and high quality of the data. A total of 3,820 questionnaires were distributed. After exclusion of samples with missing responses for key variables, 3,562 valid questionnaires were collected, yielding a valid response rate of 93.2%. Of these, 1,203 samples indicated a willingness to purchase care services.

**Table 1:** Sample size allocation.

City	Number of sample communities per city	Number of samples per community	Sample size
Shenyang	35	20	700
Dalian	23	20	460
Anshan	15	20	300
Chaoyang	12	20	240
Jinzhou	12	20	240
Huludao	12	20	240
Tieling	12	20	240
Yingkou	11	20	220
Dandong	10	20	200
Fushun	9	20	180
Fuxin	8	20	160
Liaoyang	8	20	160
Panjin	7	20	140
Benxi	7	20	140
Sum	181		3,820

Although data were collected over a four-year period (2021–2024), the same sampling frame, questionnaire, and interviewer training protocols were applied each year. We compared key demographic variables (age, gender, education, and urban/rural residence) across survey years using chi-square tests and found no significant differences (*p* > 0.05 for all comparisons). This suggests that the samples are sufficiently consistent for pooled analysis and that no major temporal biases were introduced.

### 3.2 Variable setting

#### 3.2.1 Dependent variable

The dependent variable in this study is older people’s intention to purchase home-based care services, as measured by their willingness to purchase services and the types of services they intend to purchase.

Regulations from the Chinese civil affairs departments categorize home-based care services as follows: daily living care, medical and health care, and information consultation. These categories encompass nine specific service items: home visits, older adult canteens, daily living assistance, shopping assistance, cleaning services, medical health, psychological counseling, service hotlines, and legal aid. The above three service categories were all set as binary variables, with a willingness to purchase any item within that category coded as 1 and otherwise as 0.

#### 3.2.2 Independent variables

Based on the literature review, this study takes the basic characteristics, individual needs, resource conditions, and concepts of older adult care for older people as its independent variables.

In terms of basic characteristics, the variables of gender, age, education level, marital status, and living arrangements of older people were set as independent variables.

The individual needs considered here are the physiological and psychological needs of older people. Physiological needs were assessed in terms of daily activity ability and chronic disease status. The ADL (Activities of Daily Living) scale was used to measure daily activity ability, with scores ranging from 10 to 30, and higher scores indicating greater disability. For chronic disease status, a binary response was used, with 0 = none and 1 = yes. Psychological needs were assessed in terms of cognitive ability and loneliness. A set of 16 common-knowledge questions was used to measure cognitive ability, with each question worth one point, and total scores thus ranging from 0 to 16. Loneliness was measured by the question “Do you often feel lonely?” with a score ranging from 1 to 3, with higher scores indicating greater loneliness.

Regarding resource conditions, community resources were assessed by the question “Does your community provide the following services?” with a list of nine service options. If an older person selected any one of the nine services, the community was counted as providing care services. Due to data availability constraints, we were unable to capture service quantity, quality, or frequency. Thus, we adopted this binary indicator as an initial screening measure, acknowledging its limitations. This binary indicator is a coarse measure of community resources, as it ignores the number, quality, frequency, and accessibility of services. We adopt this simplified definition as an initial exploration, but the limitations should be kept in mind when interpreting the contribution rate of community resources (45.3%). Family resources were assessed by the questions “In the past year, have your children given you money, goods, or other gifts?” and “In the past year, have your children helped you with housework?” Personal resources were measured as the respondent’s monthly pension income, with no pension income recorded as 0.

Older adult care concepts were assessed using the question “Who do you think should mainly bear the responsibility for care services?” with response options being the state, children, older people themselves, and shared responsibility.

The assignment of values for the dependent and independent variables, as well as the descriptive statistical results, are presented in Table 2.

**Table 2:** Descriptive statistics and assignment of variables.

Variable dimensions	Variables and assignments	Proportion or mean	Variable dimensions	Variables and assignments	Proportion or mean
Basic features	Gender		Individual needs (physiological)	ADL (scores)	10.53
	Female = 0	49.17%		Chronic disease status	
	Male = 1	50.83%		Without = 0	20.13%
	Age			With = 1	79.87%
	60–69 = 1	45.19%	Individual needs (psychological)	Cognitive ability (scores)	13.33
	70–79 = 2	40.87%		Loneliness (scores)	1.58
	Over 80 = 3	13.94%	Resource (community)	Provide care services	
	Educational level			No = 0	67.98%
	Elementary school or below = 1 education or below	37.24%		Yes = 1	32.02%
	Junior high school = 2	52.44%	Resource (family)	Children provide support	
	High school or above = 3	10.32%		No = 0	10.80%
	Marital status			Yes = 1	89.20%
	Not married = 0	24.27%		Children provide services	
	Married = 1	75.73%		No = 0	17.79%
	Residential arrangements			Yes = 1	82.21%
	Not living alone = 0	90.40%	Resource (Individual)	Income (1,000 yuan)	4.26
	Living alone = 1	9.60%	Concepts	Care responsibilities	
				State = 1	12.47%
				Children = 2	33.35%
				Themselves = 3	11.18%
				Shared = 4	43.00%

### 3.3 Analysis method

All statistical analyses were performed using Stata 17.0. First, descriptive statistics were computed to characterize older adults’ purchase intention for home-based care services. Second, given that the dependent variables (overall purchase intention and intentions for three service categories) were binary, binary logistic regression models were estimated. All independent variables were entered simultaneously using the enter method (forced entry), rather than stepwise selection, to avoid variable selection bias and to allow full interpretation of the subsequent Shapley decomposition. Before estimating the models, we assessed multicollinearity using the variance inflation factor (VIF); the results are reported in the Results section. Model fit was evaluated using the Hosmer–Lemeshow test and the area under the ROC curve (AUC), in addition to the Nagelkerke pseudo *R*^2^.

To compare the relative importance of the four variable groups (basic characteristics, individual needs, resource conditions, and older adult care concepts) in explaining purchase intention, we applied Shapley value decomposition. The decomposition quantifies each group’s marginal contribution to the model’s pseudo *R*^2^. To examine the robustness of the Shapley results, we performed a bootstrap analysis with 500 replications, resampling the original dataset with replacement and recomputing the contribution rates. The 95% confidence intervals for each factor group are presented in the Results section.

Finally, because multiple hypotheses were tested simultaneously (15 independent variables in the full model), we addressed the issue of multiple comparisons. A conservative Bonferroni correction was applied, setting the significance threshold at *α* = 0.05/15 ≈ 0.0033. Accordingly, in the regression tables, effects with *p* < 0.01 are considered statistically significant after correction, while those with *p* < 0.05 but > 0.0033 are interpreted as marginally significant. Readers are advised to interpret the latter with caution.

## 4 Results

### 4.1 Descriptive analysis of home-based care service purchase intention

Of the 3,562 valid respondents, 1,203 (33.77%) were willing to purchase at least one service, reflecting a moderate overall intention to purchase. Of the service items, the demand for daily living care services was the highest, accounting for 31.70%, followed by medical and healthcare services at 14.43%. The lowest demand was for information and consultation services, at 8.01%. The four services with the strongest purchase intention were older adult canteens (23.13%), cleaning services (19.01%), daily living assistance (16.31%), and medical health (14.43%). The data for the specific purchase intentions are presented in Table 3.

**Table 3:** Purchase intention of older people for care services.

Service items	Proportion with willingness to purchase services
Daily living care services	31.70%
Home visits	1.21%
Older adult canteens	23.13%
Shopping assistance	8.37%
Daily living assistance	16.31%
Cleaning services	19.01%
Medical and health care	14.43%
Medical health	14.43%
Information consultation	8.00%
Service hotlines	2.39%
Legal aid	4.46%
Psychological counseling	3.76%
Willingness to purchase at least one service	33.77%

### 4.2 Multicollinearity assessment

Before estimating the logistic models, we computed the variance inflation factor (VIF) for all independent variables. The VIF values ranged from 1.02 to 2.21, with a mean of 1.32, indicating no serious multicollinearity. The Hosmer–Lemeshow test for the full model yielded *χ*^2^ = 9.34 (df = 8, *p* = 0.31), suggesting good model fit. The area under the ROC curve (AUC) was 0.78 (95% CI, 0.76–0.80), indicating acceptable discriminative ability.

### 4.3 Influencing factors of home-based care service purchase intention

Table 4 presents the binary logistic regression results for the four categories of factors (i.e., basic characteristics, individual needs, resource conditions, and older adult care concepts,) in relation to the older people’s service purchase intention.

**Table 4:** Binary logistic regression results for influencing factors of home-based care service purchase intention.

Variables (reference groups or units)	OR (SE)
Basic features	
Gender (female)	0.920 (0.081)
Age (60–69 years)	
70–79 years	0.871(0.084)
80 years and older	0.544^***^ (0.084)
Education level (elementary school education or below)	
Junior high school	1.551^***^ (0.162)
High school	1.850^***^ (0.238)
Marital status (single)	1.141 (0.149)
Living arrangement (not living alone)	0.932 (0.178)
Individual needs	
ADL (scores)	1.116^***^ (0.027)
Chronic disease status (without)	1.319^**^ (0.165)
Cognitive ability (scores)	0.920^***^ (0.016)
Loneliness (scores)	1.127^*^ (0.074)
Resource conditions	
Community services (not provide)	4.751^***^ (0.425)
Children support (not provide)	0.973 (0.144)
Children services (not provide)	1.137 (0.157)
Income (1,000 yuan)	1.312^***^ (0.032)
Concepts	
State	
Children	0.502^***^ (0.077)
Themselves	0.910 (0.154)
Shared	1.033 (0.141)
_cons	0.058^***^ (0.029)
Nagelkerke *R*^2^	0.255
*n*	3,562

^**^p < 0.05, ^***^p < 0.01. After Bonferroni correction for 15 tests, the significance threshold is *α* = 0.0033. Thus, ^***^p < 0.01 indicates statistical significance, while ^**^p < 0.05 indicates marginal significance.

With regard to basic characteristics, respondents who were younger or had higher levels of education were more willing to purchase services. The proportion of older people aged 70–79 and 80 and above who were willing to purchase services was 54.4%, while 87.1% of those aged 60–69 were willing to do so. The proportion of older people with a junior high school education or a high school education or above who were willing to purchase services were 1.55 times and 1.85 times that of those with an elementary school education or below, respectively.

In terms of individual needs, older people with poorer self-care ability, chronic diseases, poorer cognitive ability, or stronger loneliness had a higher willingness to purchase services. In terms of physiological needs, a one-point increase in ADL score was associated with an 11.6% higher likelihood of purchase intention. The proportion of older people with chronic diseases willing to purchase services was 1.32 times higher, although this effect was only marginally significant after Bonferroni correction. The individual need (physiological) hypothesis H1a is thus supported. In terms of psychological needs, for every one-point increase in cognitive ability, the proportion willing to purchase services decreased by 8.0%, and for every one-point increase in loneliness, the proportion willing to purchase services increased by 12.7%. Thus, the poorer the cognitive and psychological health status of older people, the stronger their willingness to purchase services; the individual need (psychological) hypothesis H1b is validated.

In terms of resource conditions, older people who received community care services or care support from family members, or who had higher pension income, were more likely to purchase services. When their community could provide services, then the odds ratio of service purchase intention was 4.75 times that of the control group, indicating that community resources were positively associated with service purchase intention. The resource condition (social) hypothesis H2a is thus supported. In terms of family resources, the odds ratio of service purchase intention for older people who received care support from their children was 1.14 times that of the control group. However, there was no significant effect of provision of financial support on older people’s service purchase intention. Therefore, the resource condition (family) hypothesis H2b is only partially supported. In terms of individual resources, for every 1,000 yuan increase in pension income, the odds ratio of service purchase intention increased by 31.2%, indicating that individual resources were significantly associated with higher purchase intention; thus, the resource condition (individual) hypothesis H2c is supported.

With regard to older adult care concepts, the proportion of respondents who believed that children should bear the responsibility for older adult care who were willing to purchase services was only 50.2% of the proportion of those who believe that the state should bear the responsibility for older adult care and were willing to purchase services. Those who believed in shared responsibility were more willing to purchase services, with an odds ratio 1.19 times higher than the reference group. This study also found that older people with a self-care responsibility perception did not have a significant tendency to purchase services. A possible explanation for this is that those older people are often more independent and healthier, and they also have a certain level of consumption capacity. Therefore, the positive and negative factors affecting the purchase intention of this group cancel each other out under the combined effects of demand hindrance and resource promotion, leading to an insignificant regression coefficient. Thus, the older adult care concept hypothesis H3 proposed in this study is only partially supported.

The bootstrap results (500 replications) showed that the 95% confidence intervals for the contribution rates were: community resources [42.1, 48.5%], personal resources [27.9, 32.8%], basic characteristics [8.9, 11.5%], physiological needs [5.8, 7.8%], older adult care concepts [2.9, 4.2%], psychological needs [2.6, 4.1%], and family resources [0.4, 0.9%]. The ranking order remained unchanged across all bootstrap samples, supporting the stability of our findings.

### 4.4 Shapley value decomposition of the influencing factors of home-based care service purchase intention

To visually compare the explanatory power of the four groups of factors on older people’s purchase intention for home-based care services, the Shapley Value Decomposition method was used to decompose the contribution rates of the four groups of factors (see Table 5).

**Table 5:** Shapley value decomposition results for influencing factors of home-based care service purchase intention.

Variables	Contribution rate (%)	Ranking
Basic features	10.12	3
Individual needs (physiological)	6.77	4
Individual needs (psychological)	3.34	6
Resource conditions (community)	45.30	1
Resource conditions (family)	0.59	7
Resource conditions (individual)	30.38	2
Concepts	3.50	5

In terms of contribution to the model’s pseudo-*R*^2^, community resources showed the largest share (45.30%), suggesting that among the four factor groups considered, community resources had the highest relative importance in explaining purchase intention. This suggests that the availability of service resources in the community where older people reside can significantly influence their intention to purchase such services. Personal resources were the second most influential, with a contribution rate of 30.38%, demonstrating that economic income also plays an essential role in older people’s service purchase intention. The third most influential factor was basic characteristics, indicating that age, education level, and other factors significantly affect older people’s purchase intention and preferences. The factors ranked fourth to seventh were individual needs (physiological), older adult care concepts, individual needs (psychological), and family resources. Notably, family resources ranked the lowest, possibly because the current generation of older people has a strong sense of individual autonomy and economic independence, and they thus tend to choose and purchase services based on their own needs and preferences.

### 4.5 Influencing factors of purchase intention for different types of home-based care service

Based on overall service purchase intention, different home-based care services were categorized into daily living care, medical and healthcare, and information consultation, to further examine the influencing factors of older people’s purchase intention for various types of home-based care services (see Table 6).

**Table 6:** Regression results for influencing factors of purchase intention for different types of home-based care service (binary logistic model).

Variables (reference groups or units)	OR (SE)		
	Daily living care services	Medical and health care	Information consultation
Basic features			
Gender (female)	0.947 (0.058)	0.895 (0.079)	0.892 (0.087)
Age (60–69 years)			
70–79 years	0.833^***^ (0.056)	0.708^***^ (0.069)	0.907 (0.096)
80 years and older	0.555^***^ (0.060)	0.457^***^ (0.071)	0.503^***^ (0.092)
Education level (elementary school education or below)			
Junior high school	1.289^***^ (0.098)	1.144 (0.020)	1.138^***^ (0.021)
High school	1.319^***^ (0.135)	1.513^***^ (0.204)	1.799^***^ (0.275)
Marital status (single)	1.142 (0.102)	1.146 (0.143)	1.214 (0.173)
Living arrangement (not living alone)	0.908 (0.116)	0.799 (0.150)	1.123 (0.248)
Individual needs			
ADL (scores)	1.083^***^ (0.016)	1.140^***^ (0.020)	1.138^***^ (0.021)
Chronic disease status (without)	1.182^*^ (0.101)	1.658^***^ (0.240)	0.837 (0.111)
Cognitive ability (scores)	0.957^***^ (0.011)	0.903^***^ (0.014)	0.977 (0.017)
Loneliness (scores)	1.158^***^ (0.051)	1.031 (0.065)	1.525^***^ (0.099)
Resource conditions			
Community services (not provided)	3.947^***^ (0.251)	3.334^***^ (0.324)	3.104^***^ (0.335)
Children support (not provide)	0.878 (0.090)	1.076 (0.168)	1.829^***^ (0.365)
Children services (not provided)	1.180^*^ (0.108)	1.148 (0.165)	1.034(0.157)
Income (1,000 yuan)	1.279^***^ (0.025)	1.336^***^ (0.033)	1.134^***^ (0.032)
Concepts			
State			
Children	0.638^***^ (0.069)	0.651^***^ (0.098)	0.493^***^ (0.078)
Themselves	1.127 (0.138)	0.837 (0.146)	0.861 (0.150)
Shared	1.253^**^ (0.123)	0.867 (0.119)	0.649^***^ (0.092)
_cons	0.044^***^ (0.036)	0.016^***^ (0.119)	0.003^***^ (0.002)
*R* ^2^	0.044^***^ (0.036)	0.016^***^ (0.119)	0.003^***^ (0.002)
*n*	3,562		

^**^p < 0.05, ^***^p < 0.01. After Bonferroni correction for 15 tests, the significance threshold is *α* = 0.0033. Thus, ^***^p < 0.01 indicates statistical significance, while ^**^p < 0.05 indicates marginal significance.

In terms of basic characteristics, the promoting effect of older people’s education level on their purchase intention for information consultation services was more significant than those of daily living care and medical and health care services. The proportion of older people with a high school education or above who were willing to purchase information consultation services was 1.8 times that of those with an elementary school education or below. This was significantly higher than the purchase intention of those with a high school education or above for daily living care services (1.32 times) and medical and healthcare services (1.51 times). A possible explanation for this is that older people with higher education have a higher level of cultural literacy and rely more on professional knowledge to solve their life problems.

In terms of individual needs, older people with chronic diseases had a higher proportion who were willing to purchase daily living care services and medical and health care services, at 1.18 and 1.66 times that of those without chronic diseases, respectively. However, having chronic diseases did not significantly affect intention to purchase information consultation services. This may be because older people with chronic diseases see daily living care and medical and health care services as better suited to their living and medical needs, whereas they may feel that information consultation services do not significantly improve their quality of life. By contrast, the stronger the loneliness of older people, the higher the proportion of them who were willing to purchase information consultation and daily living care services. Specifically, for every one-point increase in loneliness, the proportion of older people willing to purchase these two types of services increased by 52.5 and 15.8%, respectively. This reflects the fact that older people generally hope to seek spiritual comfort and alleviate their inner loneliness by purchasing these services, especially information consultation services, which can provide rich communication channels that create more social interaction opportunities for older people with emotional needs. This would explain why loneliness had such a significant impact on the purchase intention of older people for these services (22). In addition, loneliness did not significantly affect purchase intention for medical and healthcare services, presumably because older people believe that such services cannot effectively alleviate their feelings of loneliness (23).

In terms of resource conditions, community care resources exerted its most significant influence on older people’s purchase intention for daily living care services, followed by medical and healthcare services, and had the least influence on information and consultation services. The occurrence ratio of older people willing to purchase these three types of services in communities that provide care services was 3.95 times, 3.33 times, and 3.10 times that of older people in communities without care services, respectively. This difference may be linked to the degree of community integration of the various services. Specifically, daily living care services are often delivered through home visits and older people use them relatively frequently, which requires the service providers to be geographically close to older people’s residences. This type of services is thus more dependent on community support. In contrast, information and consultation services are typically provided online and older people use them less frequently, making such services less dependent on the local community in terms of geographical space, resulting in a weaker promotional effect of community older adult care resources (24). Financial support from children significantly increased the occurrence ratio of older people purchasing information and consultation services, but did not significantly impact the other two types of services. This phenomenon is also likely related to the differences in service nature mentioned earlier: daily living care services and medical and health care services can more easily be provided by the labor of people in the community, whereas information and consultation services have a strong professional and technical nature and are mostly obtained through paid purchase, leading older people to prefer the use of financial support from their children to purchase information and consultation services (25).

Regarding older adult care concepts, there were significant differences in the impact of a diversified understanding of care responsibilities on older people’s intention to purchase different types of services. Compared to older people who believed in state responsibility for older adult care, those who believed in shared responsibility were more willing to purchase daily living care services, with an occurrence ratio 1.25 times higher than the former, while their willingness to purchase information and consultation services was lower, at only 64.9% the size of the reference group. These two distinct impacts may be related to the ways these two types of services are viewed by older people. Those with a diversified understanding of care responsibilities likely believe that the responsibility for daily living care should be borne mainly by themselves or their children, while the responsibility for information and consultation lies with the government and society. This difference in perceived responsibility leads to differences in the purchase intentions for the two types of services among older people.

## 5 Discussion

Based on the findings, we propose three targeted recommendations to better meet older adults’ service needs, promote home-based care services, and enhance their quality of life.

First, expand community-based service resources. Shapley decomposition shows community resources contribute most (45.3%), and older adults in communities with any service had 4.75 times higher purchase intention (*p* < 0.01). Governments should integrate existing resources, invest in community-embedded facilities (e.g., older adult canteens, day care centers), and increase service site density.

Second, raise income levels. Personal income is the second strongest contributor (30.4%). For every 1,000 yuan increase in monthly pension, purchase intention odds increase by 31.2% (*p* < 0.01). Policies should increase pension amounts and coverage, and coordinate social insurance, welfare, and subsidies.

Third, optimize the service supply structure based on differentiated needs. Demand for daily living care (31.7%) far exceeds that for medical care (14.4%) and information consultation (8.0%). Older adult canteens (23.1%) and cleaning services (19.0%) are most desired. Basic services should be expanded first, while targeted provision (e.g., information consultation for lonely older adults, OR = 1.525) should also be addressed.

Our finding that community resources are most critical (45.3%) aligns with recent Chinese studies (17), but diverges from Western studies where individual income dominates (20), likely due to China’s rapid expansion of community-embedded facilities. The strong income effect (OR = 1.312) contrasts with some European null findings (18), possibly reflecting higher out-of-pocket expenses in China. The weak role of family resources (0.59%) challenges traditional filial piety expectations but matches recent surveys showing a shift toward formal services (23).

Several limitations should be acknowledged. First, the outcome measures purchase intention, not actual behavior. Given this, the findings should be interpreted as exploratory rather than predictive of actual service uptake. Policy recommendations derived from intention data warrant cautious application until replicated with behavioral outcomes. Second, because the data are cross-sectional, the reported associations do not imply causality. Unobserved confounding variables may exist, and reverse causality cannot be ruled out. Third, community resources were measured dichotomously (presence vs. absence of any service), ignoring service quantity, quality, accessibility, and affordability. This coarse measure may have overestimated or underestimated the true effect size. Consequently, the observed odds ratio (OR ≈ 4.75) and contribution rate (45.3%) should be interpreted with caution; more refined measures might yield different magnitudes of association. Fourth, the Shapley value decomposition results depend on the set of variables included and the pseudo-*R*^2^ metric of logistic regression models. They indicate relative contributions to model explanatory power, not causal importance or real-world effect sizes beyond the model. Fifth, after Bonferroni correction (*α* = 0.0033), some effects with *p* < 0.05 but >0.0033 are only marginally significant. Readers should interpret these effects with caution. Future longitudinal and behavioral studies with refined measures of community resources (e.g., service density, user satisfaction, distance to facilities) are needed to validate and extend our findings.

## 6 Conclusion

This study uses survey data collected by a research team from Ningxia University across 14 cities in Liaoning Province from 2021 to 2024 to construct an analytical framework for the factors influencing older people’s purchase intention for home-based care services. It uses descriptive statistics, binary logistic regression, and Shapley Value Decomposition to analyse the current state of older people’s purchase intentions and their influencing factors and compares the contribution rates of different factors. Its key findings are as follows. First, more than three in 10 (33.77%) older people are willing to purchase home-based care services. Second, the basic characteristics, individual needs, resource conditions, and older adult care concepts of older people significantly impact their purchase intentions. Specifically, older people who are younger, have higher education levels, have intense individual needs (i.e., higher ADL scores, chronic diseases, poor cognitive ability, or strong feelings of loneliness), have abundant resource conditions (i.e., community provision of care services, support from children, higher pension income), or possess a diversified understanding of care responsibilities are more likely to purchase care services. Third, community resources are the most critical factor affecting older people’s purchase intentions, with a contribution rate of 45.30%. Fourth, due to the significant differences in the nature and content of the various services, factors such as education level, health status, community, and family resources also differentially affect older people’s purchase intentions for different types of services. Overall, the willingness of older people to purchase daily living care services is the highest, followed by that for medical and health care services, while that for information and consultation services is the lowest.

## Data Availability

The original contributions presented in the study are included in the article/supplementary material, further inquiries can be directed to the corresponding author.
